# Long-term real-world experience with ipilimumab and non-ipilimumab therapies in advanced melanoma: the IMAGE study

**DOI:** 10.1186/s12885-021-08032-y

**Published:** 2021-05-29

**Authors:** Stéphane Dalle, Laurent Mortier, Pippa Corrie, Michal Lotem, Ruth Board, Ana María Arance, Frank Meiss, Patrick Terheyden, Ralf Gutzmer, Brian Buysse, Kelly Oh, Jane Brokaw, T. Kim Le, Susan D. Mathias, Julie Scotto, Jennifer Lord-Bessen, Andriy Moshyk, Srividya Kotapati, Mark R. Middleton

**Affiliations:** 1 Hospices Civils de Lyon, Centre Hospitalier Lyon-Sud, 69495 Pierre-Bénite, France; 2 Université de Lille, INSERM U1189, CHRU Lille, 59037 Lille, France; 3 Cambridge University Hospitals NHS Foundation Trust, Cambridge, CB0 2QQ UK; 4 Hadassah Hebrew University Hospital, 91120 Jerusalem, Israel; 5 Royal Preston Hospital, Preston, PR2 9HT UK; 6 Hospital Clínic Barcelona, 08036 Barcelona, Spain; 7 Department of Dermatology, Faculty of Medicine, Medical Center – University of Freiburg, 79104 Freiburg, Germany; 8 University of Lübeck, 23538 Lübeck, Germany; 9 Medizinische Hochschule Hannover, 30625 Hanover, Germany; 10 Syneos Health, Morrisville, NC 27560 USA; 11 Bristol Myers Squibb, Princeton, NJ 08543 USA; 12 Health Outcomes Solutions, Winter Park, FL 32790 USA; 13 Churchill Hospital, Oxford, OX3 7DQ UK

**Keywords:** Advanced melanoma, Ipilimumab, Overall survival, Quality of life, Real-world, Subsequent therapy

## Abstract

**Background:**

Ipilimumab has shown long-term overall survival (OS) in patients with advanced melanoma in clinical trials, but robust real-world evidence is lacking. We present long-term outcomes from the IMAGE study (NCT01511913) in patients receiving ipilimumab and/or non-ipilimumab (any approved treatment other than ipilimumab) systemic therapies.

**Methods:**

IMAGE was a multinational, prospective, observational study assessing adult patients with advanced melanoma treated with ipilimumab or non-ipilimumab systemic therapies between June 2012 and March 2015 with ≥3 years of follow-up. Adjusted OS curves based on multivariate Cox regression models included covariate effects. Safety and patient-reported outcomes were assessed.

**Results:**

Among 1356 patients, 1094 (81%) received ipilimumab and 262 (19%) received non-ipilimumab index therapy (systemic therapy [chemotherapy, anti–programmed death 1 antibodies, or BRAF ± MEK inhibitors], radiotherapy, and radiosurgery). In the overall population, median age was 64 years, 60% were male, 78% were from Europe, and 78% had received previous treatment for advanced melanoma. In the ipilimumab-treated cohort, 780 (71%) patients did not receive subsequent therapy (IPI-noOther) and 314 (29%) received subsequent non-ipilimumab therapy (IPI-Other) on study. In the non-ipilimumab–treated cohort, 205 (78%) patients remained on or received other subsequent non-ipilimumab therapy (Other-Other) and 57 (22%) received subsequent ipilimumab therapy (Other-IPI) on study. Among 1151 patients who received ipilimumab at any time during the study (IPI-noOther, IPI-Other, and Other-IPI), 296 (26%) reported CTCAE grade ≥ 3 treatment-related adverse events, most occurring in year 1. Ipilimumab-treated and non-ipilimumab–treated patients who switched therapy (IPI-Other and Other-IPI) had longer OS than those who did not switch (IPI-noOther and Other-Other). Patients with prior therapy who did not switch therapy (IPI-noOther and Other-Other) showed similar OS. In treatment-naive patients, those in the IPI-noOther group tended to have longer OS than those in the Other-Other group. Patient-reported outcomes were similar between treatment cohorts.

**Conclusions:**

With long-term follow-up (≥ 3 years), safety and OS in this real-world population of patients treated with ipilimumab 3 mg/kg were consistent with those reported in clinical trials. Patient-reported quality of life was maintained over the study period. OS analysis across both pretreated and treatment-naive patients suggested a beneficial role of ipilimumab early in treatment.

**Trial registration:**

ClinicalTrials.gov, NCT01511913. Registered January 19, 2012 – Retrospectively registered, https://clinicaltrials.gov/ct2/show/NCT01511913

**Supplementary Information:**

The online version contains supplementary material available at 10.1186/s12885-021-08032-y.

## Background

According to the World Health Organization, approximately 132,000 new cases of melanoma are diagnosed worldwide each year [1]. It was estimated that 100,350 new cases of melanoma would be diagnosed and 6850 people would die from the disease in the United States in 2020 [1]. However, recent data have shown that mortality rates have decreased significantly in both males (with an annual percentage decrease of 6.9% from 2013 to 2016) and females (with an annual percentage decrease of 9.3% from 2014 to 2016) diagnosed with melanoma of the skin [2]. Treatment options for patients with unresectable or metastatic (advanced) melanoma have evolved from chemotherapy and cytokine-based therapy to immunotherapy and targeted therapy in the past decade [3]. In 2011, ipilimumab, an anti–cytotoxic T-lymphocyte antigen 4 (anti–CTLA-4) antibody, became the first approved immune checkpoint inhibitor after demonstrating significant improvement in overall survival (OS) in patients with advanced melanoma in randomised clinical trials [4–6]. Subsequently, anti–programmed death 1 (anti–PD-1) antibodies (nivolumab and pembrolizumab) as well as *BRAF* targeted therapies (vemurafenib ± cobimetinib, dabrafenib ± trametinib, and encorafenib ± binimetinib) were approved, radically improving outcomes for patients with advanced melanoma [3, 7]. More recently, nivolumab, pembrolizumab, and dabrafenib ± trametinib have shown benefits in recurrence-free survival as adjuvant treatment for high-risk resected melanoma [8].

Long-term survival has been reported in patients with advanced melanoma treated with ipilimumab in phase II and III clinical trials [9, 10]. A pooled analysis of 10 prospective and 2 retrospective studies of ipilimumab demonstrated 3-year OS of 22% for all patients, 20% in previously treated patients, and 26% in treatment-naive patients [11]. Any-grade and grade 3/4 treatment-related adverse events (TRAEs; according to National Cancer Institute Common Terminology Criteria for Adverse Events [CTCAE]) were reported in 80 and 23% of patients, respectively [4]. Although efficacy and safety results from randomised controlled trials of ipilimumab are available, data from long-term real-world studies are lacking. Real-world studies are being increasingly used to complement results from clinical trials as they represent patients who are diagnosed and treated routinely, including those who did not meet the selection criteria for registration into randomised controlled trials (e.g., patients with Eastern Cooperative Oncology Group performance status [ECOG PS] ≥ 2 or active/untreated brain metastases) [12, 13]. Since the introduction of anti–PD-1 antibodies, which have shown superior first-line efficacy compared with ipilimumab [14, 15], ipilimumab has been less commonly used as first-line monotherapy. However, it is still used in combination with nivolumab as first-line therapy in patients with advanced melanoma [14] and as subsequent therapy in patients with disease progression after single-agent anti–PD-1 treatment [16].

IMAGE (ipilimumab: management of advanced melanoma in real practice; NCT01511913) is a multinational observational study evaluating real-world treatment and outcomes for patients with advanced melanoma. We have previously published results of the retrospective IMAGE study group [17], which provided insights into patient care for advanced melanoma in the era before ipilimumab was available. Here, we present results of the prospective study group in which patients received ipilimumab or non-ipilimumab systemic therapies. We report the estimated incidence and severity of TRAEs in patients treated with ipilimumab in the postapproval setting using CTCAE criteria. We also describe OS in the overall patient population, in previously treated patients, and in treatment-naive patients treated with ipilimumab or non-ipilimumab therapies. In addition, we assess long-term quality of life (QoL) in the overall patient population.

## Methods

### Patients and study design

IMAGE is a phase IV, multinational observational study that recruited patients with advanced melanoma. The study included retrospective and prospective groups. The retrospective group consisted of patients with advanced melanoma who were treated with non-ipilimumab therapy within the 3 years prior to the approval of ipilimumab [17], whereas the prospective group consisted of patients who were enrolled once ipilimumab was approved and available for routine use. Results of the prospective group are reported here. In the prospective group, eligible patients were recruited from 200 sites across 15 countries (Argentina, Australia, Austria, Belgium, Canada, France, Germany, Greece, Ireland, Israel, Poland, Spain, Switzerland, the United Kingdom, and the United States). Patients were enrolled over a period of approximately 30 months (June 2012 to March 2015) and followed for a minimum of 3 years (Additional file 1: Fig. A.1) or until loss to follow-up, withdrawal of consent, or death. Enrollment began once ipilimumab was approved in each country and available for treatment in patients with advanced melanoma. This study included two prospective groups: patients who received ipilimumab as the index therapy (ipilimumab-treated group) and those who received any approved treatment other than ipilimumab as the index therapy (non-ipilimumab–treated group). Ipilimumab was administered at 3 mg/kg by intravenous infusion for 90 min every 3 weeks for 4 doses or until disease progression, unacceptable toxicity, or withdrawal of consent. Radiotherapy was allowed with ipilimumab as a concomitant therapy. Non-ipilimumab therapies included systemic therapy (chemotherapy, anti–PD-1 antibodies, or BRAF ± MEK inhibitors), radiotherapy, and radiosurgery.

Patients included in this study were adults (≥ 18 years of age at study entry) who were diagnosed with and treated for advanced melanoma by a health care provider (community-based, office-based, hospital-based, or academic setting). Patients who participated in another clinical trial evaluating therapy for any cancer (including advanced melanoma) or received therapy for a primary cancer other than melanoma were excluded.

This study was approved by the institutional review board/ethics committee at each participating centre (Additional file 2) and was conducted in accordance with the Declaration of Helsinki and International Society for Pharmacoepidemiology Guidelines for Good Epidemiology Practices. Patients generally provided written consent for enrollment, but they could have provided verbal consent documented by the site staff if in accordance with local regulations and the institutional review board/ethics committee.

### Analysis populations

**Fig. 1:**
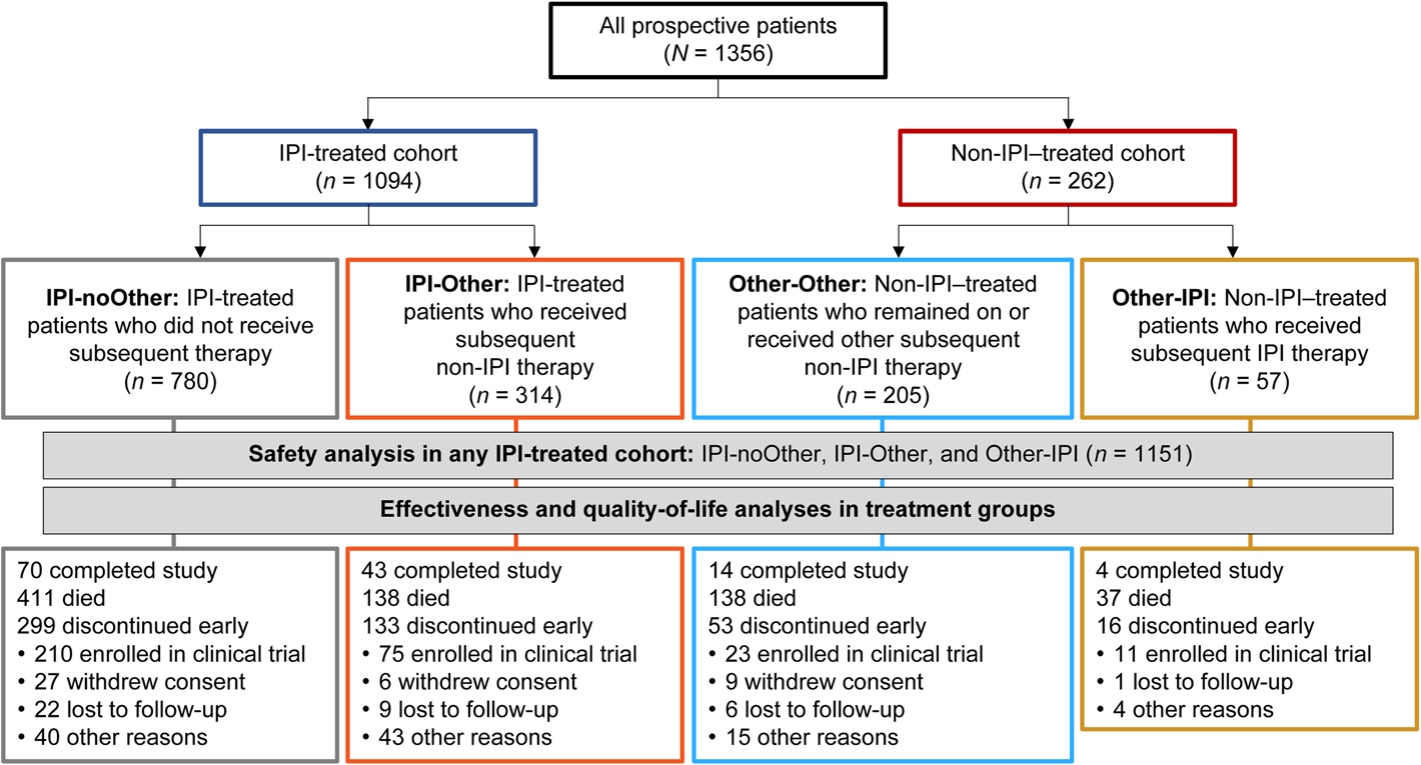
Patient disposition. *IPI* ipilimumab

Patients who received ipilimumab or non-ipilimumab therapy as index therapy were further divided into the following groups: IPI-noOther, ipilimumab-treated patients (with or without concomitant radiotherapy) who did not receive subsequent systemic therapy during the study period; IPI-Other, ipilimumab-treated patients who received subsequent systemic non-ipilimumab therapy during the study period; Other-Other, non-ipilimumab–treated patients who remained on non-ipilimumab index therapy or received other subsequent systemic non-ipilimumab therapy during the study period; and Other-IPI, non-ipilimumab–treated patients who received subsequent systemic ipilimumab therapy during the study period (Fig. 1).

Safety analysis was conducted in patients who received ipilimumab therapy (as index therapy or subsequent therapy) at any time during the study period (IPI-noOther, IPI-Other, and Other-IPI) (Fig. 1). TRAEs were not reported in patients who did not receive ipilimumab during the study period. Effectiveness and QoL assessments were conducted in all four subgroups of patients.

### Data collection

Data on patient demographics, baseline characteristics, subsequent systemic therapy, and incidence of adverse events were collected from electronic case report forms. Safety data included TRAEs (non-serious and serious), immune-related adverse events, and TRAEs leading to discontinuation or death. A serious adverse event included any medical occurrence that resulted in death, a life-threatening condition, hospitalisation or prolongation of existing hospitalisation, persistent or significant disability/incapacity, or a congenital anomaly or medical event that led to these events. Early-onset adverse events were defined as adverse events occurring between the first dose and 90 days after the last dose of ipilimumab therapy. Late-onset adverse events were defined as adverse events occurring 90 days after the last dose of ipilimumab [18]. The severity of adverse events was graded according to CTCAE v3.0.

Effectiveness was assessed using 3-year OS rates and tumor response. In this study, tumor response was based on the last tumor assessment recorded with a non-missing assessment date during the on-study period and was determined by investigators using various response criteria (the World Health Organization criteria, Response Evaluation Criteria in Solid Tumors, or other response criteria).

For QoL assessments, data were collected from patient-reported questionnaires via electronic case report forms. Patients completed the European Organisation for Research and Treatment of Cancer (EORTC) QLQ-C30 questionnaire every 3 months throughout the study period. The EORTC QLQ-C30 is a 30-item, self-completed, multidimensional, cancer-specific QoL questionnaire comprising a global health status/QoL scale, three symptom scales, six single-item scales, and five functional scales [19]. EORTC QLQ-C30 scores were scaled from 0 to 100, with higher scores representing higher global health status, greater symptom/financial burden, and higher level of functioning. A change of 10 points was considered a clinically meaningful difference [20]. Completion rates were calculated from the number of questionnaires returned from eligible patients. Mean changes in patient-reported outcome scores from baseline were evaluated descriptively for each scale; interpretations were drawn from time points that had ≥10 patients completing assessments per treatment group.

### Statistical analysis

Patient demographics and baseline characteristics were reported using descriptive statistics, including median, minimum, and maximum for continuous variables, and count and percentage for categorical variables. Descriptive data were provided for TRAEs that occurred in patients treated with ipilimumab therapy at any time during the study period. Given the unique safety profile of ipilimumab related to its distinct mechanism of action, no safety comparisons were made between ipilimumab-treated and non-ipilimumab–treated patients. Incidence rates for TRAEs were calculated by dividing the number of events by the overall total exposure during the specified time at risk. Confidence intervals (CIs) were calculated for incidence rates.

OS probabilities were estimated using the Kaplan–Meier product-limit method and adjusted based on multivariate Cox regression models that included covariate effects. Among a total of 25 covariates, 6 were selected based on a stepwise Cox regression model (Additional file 1: Table A.1). These covariates were ECOG PS at study entry, lactate dehydrogenase (LDH) status, EORTC QLQ-C30 QoL (based on overall health status score and QoL in the past week), prior therapy, sex, and Work Productivity and Activity Questionnaire change in level of responsibility at work. Normal or elevated LDH status were based on local laboratory assessments. For patient-reported outcomes, mean changes from baseline in assessment points throughout the study were evaluated for all treatment cohorts.

## Results

### Patient characteristics and treatment patterns

Among 1356 patients prospectively enrolled in this study, 1094 (81%) received ipilimumab and 262 (19%) received non-ipilimumab treatment as index therapy (Fig. 1). In the overall population, median age was 64 years, 60% of patients were male, 78% were from Europe, 78% had received previous treatment with systemic therapy for advanced melanoma, 47% had stage IV M1C disease, and 21% had CNS metastases (Table 1). Although pretreatment LDH and ECOG PS data were not assessed or missing in a substantial proportion of patients, 39% of patients had elevated LDH and 6% had ECOG PS ≥ 2 (Table 1). Among patients treated with ipilimumab therapy at any time during the study period (*n* = 1151), 719 (62%) received 4 doses (Additional file 1: Table A.2); patients received 2.5 to 3.5 mg/kg of ipilimumab, consistent with the recommended dose of 3 mg/kg.

**Table 1:** Patient demographics and baseline characteristics for all prospective patients based on index therapy

	All prospective patients (***N*** = 1356)	IPI-treated cohort		Non-IPI–treated cohort	
		IPI-noOther (***n*** = 780)	IPI-Other (***n*** = 314)	Other-Other (***n*** = 205)	Other-IPI (***n*** = 57)
Median age, years (range)	64 (22–90)	65 (22–90)	62 (25–88)	63 (24–89)	60 (27–84)
Sex, no. (%)					
Male	819 (60)	483 (62)	178 (57)	118 (58)	40 (70)
Female	537 (40)	297 (38)	136 (43)	87 (42)	17 (30)
Race, no. (%)					
White	1300 (96)	746 (96)	300 (96)	200 (98)	54 (95)
Asian	3 (< 1)	2 (< 1)	0	0	1 (2)
Other	7 (1)	6 (1)	1 (< 1)	0	0
Not specified	46 (3)	26 (3)	13 (4)	5 (2)	2 (4)
Geographic region, no. (%)					
Europe	1061 (78)	641 (82)	231 (74)	149 (73)	40 (70)
North America	170 (13)	67 (9)	64 (20)	29 (14)	10 (18)
Rest of world	125 (9)	72 (9)	19 (6)	27 (13)	7 (12)
Time on study,^a^ months					
Mean (SD)	10 (11)	9 (10)	14 (11)	9 (10)	13 (9)
Median (range)	6 (0–50)	5 (0–46)	10 (1–39)	5 (0–38)	9 (2–50)
Previous systemic therapy for advanced melanoma, no. (%)					
Yes	1064 (78)	619 (79)	233 (74)	169 (82)	43 (75)
No	292 (22)	161 (21)	81 (26)	36 (18)	14 (25)
*BRAF* mutation status, no. (%)					
Mutant	542 (40)	262 (34)	135 (43)	116 (57)	29 (51)
Wild-type	743 (55)	471 (60)	162 (52)	84 (41)	26 (46)
Inconclusive/unknown	12 (1)	8 (1)	3 (1)	1 (< 1)	0
Missing	59 (4)	39 (5)	14 (4)	4 (2)	2 (4)
ECOG PS, no. (%)					
0	470 (35)	274 (35)	134 (43)	47 (23)	15 (26)
1	328 (24)	208 (27)	59 (19)	48 (23)	13 (23)
2	63 (5)	35 (4)	5 (2)	22 (11)	1 (2)
3	13 (1)	3 (< 1)	0	8 (4)	2 (4)
4	2 (< 1)	1 (< 1)	0	1 (< 1)	0
Missing	480 (35)	259 (33)	116 (37)	79 (39)	26 (46)
Disease stage, no. (%)					
Stage III	44 (3)	26 (3)	10 (3)	3 (1)	5 (9)
Stage IV	1312 (97)	754 (97)	304 (97)	202 (99)	52 (91)
Metastases stage, no. (%)					
M0	43 (3)	25 (3)	10 (3)	3 (1)	5 (9)
M1a	150 (11)	85 (11)	41 (13)	22 (11)	2 (4)
M1b	247 (18)	144 (18)	69 (22)	29 (14)	5 (9)
M1c	638 (47)	382 (49)	143 (46)	85 (41)	28 (49)
CNS	278 (21)	144 (18)	51 (16)	66 (32)	17 (30)
CNS metastases,^b^ no. (%)					
Symptomatic	85 (31)	42 (29)	16 (31)	22 (33)	5 (29)
Asymptomatic	193 (69)	102 (71)	35 (69)	44 (67)	12 (71)
LDH status at study entry, no. (%)					
Normal	542 (40)	298 (38)	159 (51)	64 (31)	21 (37)
Elevated or outside of normal	531 (39)	310 (40)	105 (33)	92 (45)	24 (42)
Not assessed	283 (21)	172 (22)	50 (16)	49 (24)	12 (21)

*CNS* central nervous system, *ECOG PS* Eastern Cooperative Oncology Group performance status, *IPI* ipilimumab, *LDH* lactate dehydrogenase, *SD* standard deviation.

^a^Defined as the time from study completion/discontinuation/death/data cut date (whichever came first) − the index therapy date + 1; study time for a patient may have exceeded 1065 days

^b^The number of patients who reported the CNS as the site of distant metastasis was used as the denominator

In the ipilimumab-treated cohort, 780 (71%) patients did not receive subsequent therapy (IPI-noOther) and 314 (29%) received subsequent non-ipilimumab therapy (IPI-Other) during the study period. In the non-ipilimumab–treated cohort, 205 (78%) patients remained on the same non-ipilimumab therapy or received other non-ipilimumab therapy (Other-Other) and 57 (22%) received subsequent ipilimumab therapy (Other-IPI) during the study period (Fig. 1). Overall, subsequent systemic therapy was received by 313 of 1094 (29%) patients in the ipilimumab-treated cohort and 111 of 262 (42%) patients in the non-ipilimumab–treated cohort (Table 2). The most common systemic therapy was anti–PD-1 treatment (51%) in the IPI-Other group and chemotherapy (16%) in the Other-Other group.

**Table 2:** Subsequent therapies

	IPI-treated cohort		Non-IPI–treated cohort	
	IPI-noOther (***n*** = 780)^**a**^	IPI-Other (***n*** = 314)	Other-Other (***n*** = 205)	Other-IPI (***n*** = 57)
Systemic therapy, no. (%)	0	313 (100)	54 (26)	57 (100)
Immunotherapy	0	181 (58)	18 (9)	57 (100)
Anti–PD-1 agent^b^	0	161 (51)	18 (9)	10 (18)
Anti–CTLA-4 agent^c^	0	34 (11)	0	57 (100)
Other systemic therapy, no. (%)	0	186 (59)	43 (21)	15 (26)
BRAF ± MEK inhibitor^d^	0	103 (33)	18 (9)	8 (14)
Chemotherapy^e^	0	110 (35)	33 (16)	12 (21)
Other investigational agent^f^	0	11 (4)	1 (<1)	0
Other^g^	0	11 (4)	0	0
Radiotherapy^h^	138 (18)	101 (32)	21 (10)	16 (28)

*CTLA-4* cytotoxic T-lymphocyte antigen 4, *IPI* ipilimumab, *PD-1* programmed death 1.

^a^Radiotherapy was allowed with ipilimumab in this cohort; therefore, it was not considered subsequent therapy

^b^Pembrolizumab or nivolumab

^c^Ipilimumab

^d^Dabrafenib ± trametinib or vemurafenib ± cobimetinib

^e^Bleomycin, carboplatin, cisplatin, combinations of antineoplastic agents, cyclophosphamide, dacarbazine, dactinomycin, docetaxel, etoposide, fotemustine, gemcitabine, lomustine, melphalan, paclitaxel, paclitaxel + carboplatin, temozolomide, treosulfan, trofosfamide, vinblastine, vincristine, vindesine, or vinorelbine

^f^Bevacizumab or imatinib

^g^Aldesleukin, antineoplastic and immunomodulating agents, interferon-alpha, interleukin-2, melanoma vaccine, other therapeutic products, or monoclonal antibodies

^h^Radiation, radiosurgery, radiotherapy, or yttrium (^90^Y)

### Safety

Safety was assessed in patients who had received the first dose of ipilimumab at any time during the study period as index therapy or subsequent therapy (IPI-noOther, IPI-Other, and Other-IPI; *n* = 1151). TRAEs of any grade and of grade ≥ 3 were reported in 66% (756 of 1151) and 26% (296 of 1151) of patients, respectively (Table 3). Early-onset TRAEs of any grade were reported in 64% (734 of 1151) of patients, and grade ≥ 3 events were reported in 24% (275 of 1151) of patients. Among those who were followed post-treatment, late-onset TRAEs of any grade were reported in 20% (128 of 653) of patients and were grade ≥ 3 in 6% (42 of 653) of patients. The majority of TRAEs were consistent with the mechanism of action of ipilimumab; the most common any-grade TRAEs were diarrhoea (21%), fatigue (16%), rash (12%), and nausea (10%) (Additional file 1: Table A.3). TRAEs or serious adverse events that led to death were reported in 22 patients; of these, 10 were attributed to immune-related adverse events. For many of these reported deaths, the cause was also listed as “succumbed to melanoma” or as unknown; therefore, whether these deaths were due to TRAEs or whether the adverse events were ongoing at the time of death, but not the direct cause, is unclear. Immune-related adverse events of any grade and grade ≥ 3 were reported in 49 and 19% of patients, respectively (Additional file 1: Table A.4). Overall, the incidence of grade ≥ 3 ipilimumab-related adverse events was higher in the first year compared with subsequent years after ipilimumab treatment initiation (Additional file 1: Table A.5).

**Table 3:** Summary of adverse events using CTCAE criteria

	All IPI-treated patients^**a**^		
	On study^**b**^ (***n*** = 1151)	Early onset^**c**^ (***n*** = 1151)	Late onset^**d**^ (***n*** = 653)
Any treatment-related adverse event, no. (%)			
Any grade	756 (66)	734 (64)	128 (20)
Grade ≥ 3	296 (26)	275 (24)	42 (6)
Serious adverse event	225 (20)	218 (19)	15 (2)
Immune-related adverse event, no. (%)			
Any grade	569 (49)	550 (48)	65 (10)
Grade ≥ 3	211 (19)	211 (18)	20 (3)
Treatment-related adverse event/serious adverse event leading to discontinuation, no. (%)			
Any grade	158 (14)	148 (13)	14 (2)
Grade ≥ 3	102 (9)	99 (9)	4 (1)
Treatment-related adverse event/serious adverse event leading to death,^e^ no. (%)			
Any grade	22 (2)	21 (2)	1 (<1)
Grade ≥ 3	17 (1)	16 (1)	1 (<1)

*CTCAE* National Cancer Institute Common Terminology Criteria for Adverse Events, *IPI,* ipilimumab.

^a^Includes patients who received ipilimumab therapy at any time during the study period (IPI-noOther, IPI-Other, Other-IPI)

^b^From first dose of ipilimumab until discontinuation from study or end of study, whichever came first

^c^Occurring between the first dose and 90 days after the last dose of ipilimumab therapy

^d^Occurring 90 days after the last dose of ipilimumab; the denominator is the number of ipilimumab-treated patients who were in the post-treatment phase of follow-up

^e^10 deaths were attributed to immune-related adverse events

### Effectiveness

**Fig. 2:**
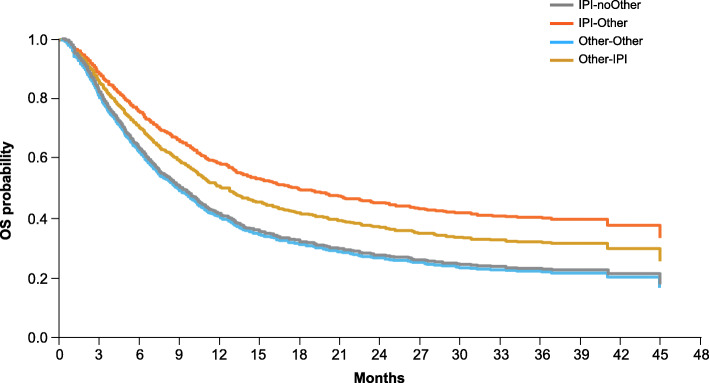
OS in the ipilimumab-treated and non-ipilimumab treated cohorts. *IPI* ipilimumab, *OS* overall survival

The 3-year OS rate was 28% in the ipilimumab-treated cohort and 25% in the non-ipilimumab–treated cohort (Additional file 1: Fig. A.2). In a multivariate model in which the reference subgroup was IPI-noOther, the hazard ratio was 0.57 (95% CI 0.47 to 0.68; *p*<0.001) for IPI-Other, 1.01 (95% CI 0.83 to 1.23; *P* = 0.897) for Other-Other, and 0.73 (95% CI 0.53 to 1.01; *P* = 0.061) for Other-IPI (Fig. 2). Patients in the IPI-Other group had a 43% reduced risk of death compared with those in the IPI-noOther group. Among the treatment subgroups, ipilimumab-treated and non-ipilimumab–treated patients who switched therapy (IPI-Other and Other-IPI) had longer OS than those who did not switch therapy (IPI-noOther and Other-Other). Patients in the IPI-Other group tended to have longer OS than those in the Other-IPI group (Fig. 2).

**Fig. 3:**
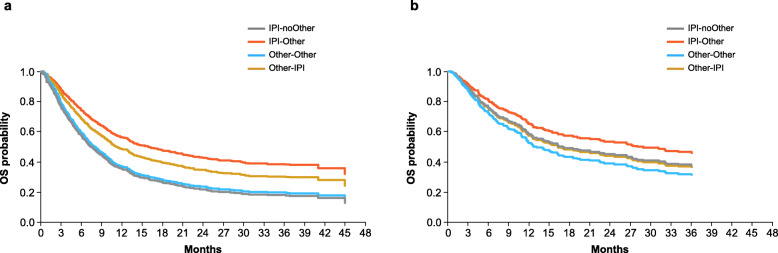
OS in previously treated **a** and treatment-naive **b** patients in ipilimumab-treated and non-ipilimumab–treated cohorts. *IPI* ipilimumab, *OS* overall survival

In previously treated patients (*n* = 1064), 3-year OS rates were 25% in the ipilimumab-treated cohort and 22% in the non-ipilimumab–treated cohort; the OS rates among treatment-naive patients (*n* = 292) were 40 and 33%, respectively (Additional file 1: Fig. A.3). In multivariate models comparing the four subgroups, the IPI-noOther group was considered the reference subgroup. For previously treated patients, the hazard ratio was 0.53 (95% CI 0.43 to 0.65; *P* < 0.001) for IPI-Other, 0.98 (95% CI 0.80 to 1.21; *P* = 0.882) for Other-Other, and 0.68 (95% CI 0.47 to 0.99; *P* = 0.045) for Other-IPI (Fig. 3a). Patients with prior therapy who did not switch therapy (IPI-noOther and Other-Other) showed similar OS. For treatment-naive patients, the hazard ratio was 0.77 (95% CI 0.51 to 1.16; *P* = 0.219) for IPI-Other, 1.21 (95% CI 0.72 to 2.01; *P* = 0.473) for Other-Other, and 1.04 (95% CI 0.50 to 2.15; *P* = 0.926) for Other-IPI (Fig. 3b). Patients in the IPI-noOther group had a 21% reduced risk of death (not significant) compared with those in the Other-Other group.

Disease control rates (the sum of the rates for complete response, partial response, and stable disease) were 16% for IPI-noOther, 20% for IPI-Other, 16% for Other-Other, and 7% for Other-IPI (Additional file 1: Table A.6).

### Quality of life

**Fig. 4:**
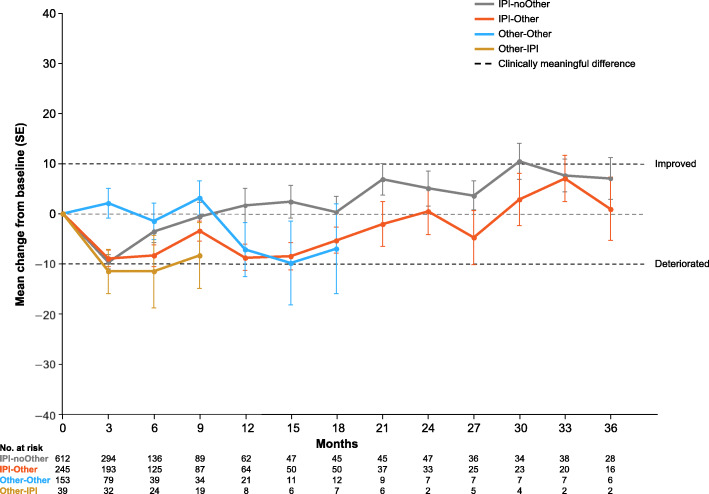
Mean changes from baseline on treatment for EORTC QLQ-C30 global health status. *EORTC* European Organisation for Research and Treatment of Cancer, *IPI* ipilimumab, *QLQ*-*C30* Core Quality of Life Questionnaire, *SE* standard error

In the overall patient population, completion rates for EORTC QLQ-C30 global health status were between 58 and 80% (Additional file 1: Table A.7). On treatment, there were no major differences between the four subgroups in mean changes from baseline for EORTC QLQ-C30 global health status (Fig. 4). Among surviving patients in all treatment groups, initial global health status changes from baseline were mostly negative (indicating deterioration) until approximately year 2 and became positive (indicating improvement) by year 3. Notable exceptions were observed in IPI-noOther group, in which patients showed improvement by the end of year 1, and for Other-Other group, in which patients did not show deterioration until the end of year 1. Numerically, the three symptom scales (fatigue, nausea and vomiting, and pain), six single-item symptom scores (dyspnea, insomnia, appetite loss, constipation, diarrhea, and financial difficulties), and four of the five functional scales (physical, role, cognitive, and social) generally showed either no change or deterioration from baseline early in the study. Improvement by the end of year 3 resulted in changes that were not clinically meaningful (Additional file 1: Fig. A.4a–m). However, the emotional functional scale showed primarily positive changes (indicating improvement) throughout the study (Additional file 1: Fig. A.4n).

## Discussion

This prospective real-world study presents long-term outcomes (≥ 3 years of follow-up) of patients with advanced melanoma treated with ipilimumab and other therapies. Safety outcomes and OS in patients treated with ipilimumab 3 mg/kg were similar to those previously reported in ipilimumab randomised clinical trials, despite the inclusion of patients who are typically excluded from randomised controlled trials (e.g., those with ECOG PS ≥ 2 or active/untreated brain metastases) [12, 13]. This study also showed that patients who switched therapy (from ipilimumab or non-ipilimumab therapies) had longer OS than those who did not switch therapy. QoL did not deteriorate significantly over the study period in either treatment cohort.

The patient population in the IMAGE study was broadly comparable to those reported in ipilimumab randomised clinical trials [4–6], and other real-world studies [21, 22]. The types, incidence, and severity of all adverse events associated with ipilimumab were similar to those previously reported in patients with advanced melanoma. No new safety signals were identified. In phase III trials, TRAEs of any grade and grade ≥ 3 were reported in 63 to 80% and 18 to 23% of patients treated with ipilimumab 3 mg/kg, respectively [4, 6]; in the current study these rates were 66 and 26%, respectively, mostly immune-related. Even though 66% of patients experienced TRAEs, most (62%) were able to complete the four recommended infusions of ipilimumab.

The proportion of ipilimumab-treated patients who received subsequent systemic therapy (29%) in this study was lower than that observed in a phase III clinical trial comparing different doses of ipilimumab (38%) [6], perhaps due to differences between the real-world population included in the current study and those included in clinical trials with stringent entry criteria or differences in the availability of subsequent therapies at the time when these studies were conducted. In the current study, a higher proportion of patients in the non-ipilimumab–treated cohort received subsequent systemic therapy than those in the ipilimumab-treated cohort (42% vs. 29%). Recent trials have shown that a higher frequency of subsequent treatment use is associated with less efficacious initial therapy [14, 23].

OS results from the current study were consistent with those observed in prospective clinical trials of patients with advanced melanoma receiving ipilimumab. In a pooled analysis, the 3-year OS rate was 22% for all patients, 20% for previously treated patients, and 26% for treatment-naive patients [11]; in the IMAGE study these rates were 28, 25, and 40%, respectively. The higher OS rates in the current study may be attributed to the changing treatment landscape for advanced melanoma, which evolved rapidly during the period in which this study was conducted (2012 to 2015). Initially, ipilimumab was the only approved immune checkpoint inhibitor that was available for treatment of patients with advanced melanoma. Subsequently, dabrafenib (targeted therapy) was approved in 2013, and pembrolizumab and nivolumab (anti–PD-1 agents) were approved in 2014, becoming available for use as subsequent therapies. Availability of these newer agents may have contributed to longer OS in patients who switched from ipilimumab or non-ipilimumab therapy than those who did not switch therapy. Similar improvements in OS were observed in prospective trials in which patients who had disease progression after ipilimumab therapy received subsequent anti–PD-1 treatment [24, 25]. Of note, OS in the IMAGE study was similar between the IPI-Other and Other-IPI cohorts, and OS was longer in both of those cohorts than in the IPI-noOther and the Other-Other cohorts, suggesting that the use of immune checkpoint inhibitor therapy in sequence with other therapies may provide superior survival outcomes compared with other regimens. The potential residual effect of immortal time bias (also known as survival treatment selection bias) should be considered in interpreting the magnitude of difference in OS between cohorts in which patients switched or did not switch therapy.

Although the treatment paradigm for metastatic melanoma has shifted with the use of anti–PD-1 checkpoint inhibitors alone or in combination with ipilimumab, ipilimumab monotherapy may still be a consideration, such as in the treatment of particular patient subgroups following failure of anti–PD-1 therapy [14]. Some clinical trials have shown that ipilimumab has antitumour activity in patients who had disease progression on anti–PD-1 therapy and received subsequent ipilimumab [26, 27]. In the current study, patients in the Other-IPI group tended to have longer survival than those in the Other-Other group. Descriptive OS analysis of patients who were previously treated and those who were treatment-naive suggested a beneficial role for ipilimumab as first-line treatment for patients with advanced melanoma in an era when anti–PD-1 therapies were not available.

During 3 or more years of follow-up, no major differences in QoL outcomes were observed between the four ipilimumab and non-ipilimumab–treated cohorts. This observation is consistent with the results reported in a phase 3 clinical trial in patients with advanced melanoma in which ipilimumab did not have a significant negative impact on health-related QoL during the treatment induction phase [28]; however, long-term QoL results were not available for that trial. Although the current analysis did show initial deterioration in global health status (with improvement by year 3), these findings are difficult to correlate with disease progression, toxicities, or other clinical outcomes and would benefit from further research. There was no change or deterioration from baseline in most of the QLQ-C30 components (three symptom scales, six single-item symptom scores, and four of five functional scale scores [physical, role, cognitive, and social]), with improvements by the end of year 3. However, these results should be interpreted with caution as patients who completed the questionnaires were more likely to have been doing better than those who did not; moreover, the cohorts that started with a non-ipilimumab treatment had considerably fewer patients than the ipilimumab-treated cohorts.

This study had limitations, largely those inherent to a real-world study, that should be considered in interpreting the findings. Data were limited in a number of clinical characteristic categories because of incomplete patient information. For example, ECOG PS was missing for 33 to 46% of patients, LDH status was not assessed in 16 to 24% of patients, and *BRAF* status was unknown for 2 to 6% of patients. In addition, the patient population was not uniform between the study cohorts because of the non-randomised nature of the study. For example, more patients had central nervous system metastasis in the non-ipilimumab–treated cohort (30 to 32%) than in the ipilimumab-treated cohort (16 to 18%).

## Conclusions

Long-term, real-world safety and effectiveness in the IMAGE study were consistent with safety and efficacy in ipilimumab clinical trials. OS analysis across previously treated and treatment-naive patients suggested a beneficial role of ipilimumab early in the disease, with no detrimental impact on QoL. Although ipilimumab is no longer commonly used as first-line monotherapy for patients with advanced melanoma, the results of this study provide reassurance that patients who switch to ipilimumab still gain benefit and that the safety profile of the drug in clinical trials is not significantly different in real-world clinical practice.

## Supplementary Information

**Additional file 1: Supplement. Table A.1.** Covariates evaluated in the multivariate Cox regression models for OS probabilities. **Table A.2.** Ipilimumab dosing. **Table A.3.** Summary of treatment-related adverse events in patients who received ipilimumab therapy at any time during the study period (includes groups 1, 2, and 4).** Table A.4.** Summary of immune-related adverse events in patients who received ipilimumab therapy at any time during the study period (includes groups 1, 2, and 4). **Table A.5.** Incidence rate of treatment-related adverse events of grade ≥ 3 in patients who received ipilimumab therapy at any time during the study period (includes groups 1, 2, and 4).** Table A.6.** Tumor response.** Table A.7**. Patient-reported outcome completion rates.** Fig. A.1.** Study design.** Fig. A.2.** OS in the ipilimumab-treated and non-ipilimumab-treated cohorts. *P* value was estimated from type 3 Wald test from the Cox model. *OS* Overall survival.** Fig. A.3.** OS in (A) previously treated (*n* = 1064) and (B) treatment-naive (*n* = 292) patients. *P* values were estimated from type 3 Wald test from the Cox model. *OS* overall survival.** Fig. A.4.** Patient-reported outcomes in three symptom scales (fatigue, nausea and vomiting, and pain), six single-item symptom scores (dyspnea, insomnia, appetite loss, constipation, diarrhea, and financial difficulties), and five functional scales (physical, role, cognitive, social, and emotional functioning)

**Additional file 2.** Institutional review boards/ethics committees.

## Data Availability

Data underlying the findings described in this manuscript may be obtained in accordance with Bristol Myers Squibb’s data sharing policy described at: https://www.bms.com/researchers-and-partners/independent-research/data-sharing-request-process.html.

## Abbreviations

anti–CTLA-4: Anti–cytotoxic T-lymphocyte antigen 4
anti–PD-1: Anti–programmed death 1
CI: Confidence interval
CTCAE: National Cancer Institute Common Terminology Criteria for Adverse Events
ECOG PS: Eastern Cooperative Oncology Group performance status
EORTC: European Organisation for Research and Treatment of Cancer
LDH: Lactate dehydrogenase
OS: Overall survival
QoL: Quality of life
TRAE: Treatment-related adverse event
