# Supplementary material for: Long-term real-world experience with ipilimumab and non-ipilimumab therapies in advanced melanoma: the IMAGE study
Source: BMC Cancer. 2021 May 29;21:642. doi: 10.1186/s12885-021-08032-y (PMC8164785; doi:10.1186/s12885-021-08032-y)

**Long-term real-world experience with ipilimumab and non-ipilimumab therapies in advanced melanoma: the IMAGE study**

**SUPPLEMENTARY APPENDIX**

**Table A.1** Covariates evaluated in the multivariate Cox regression models for OS probabilities^a^

| **Covariate** | **Categories** |
| --- | --- |
| Age | Years |
| Sex | Female vs male |
| Disease stage at study entry | Stage III vs IV |
| Disease stage at initial diagnosis | Stage I, II, III vs IV |
| Metastatic status at study entry | Yes vs no |
| *BRAF V600* mutation status | Yes; no (referent); inconclusive/unknown due to technical issues |
| LDH status | Normal (referent); elevated or outside of range; not done |
| Treatment status at study entry | Pre-treated vs treatment naïve |
| Prior systemic therapy | Yes vs no |
| Prior radiotherapy | Yes vs no |
| Prior radiosurgery | Yes vs no |
| EORTC QLQ-30 quality of life | Below median score; median score or above; not done/missing |
| WPAI-GH absence at work | Employed, unchanged; employed, changed; unemployed; not done/missing |
| WPAI-GH presence at work | Employed, unchanged; employed, changed; unemployed; not done/missing |
| WPAI-GH productively loss at work | Employed, unchanged; employed, changed; unemployed; not done/missing |
| WPAI-GH impaired activity | Below median score; median score or above; not done/missing |
| WPAQ change in activity at work | Employed, unchanged; employed, changed; unemployed; not done/missing |
| WPAQ change in emotional stress at work | Employed, unchanged; employed, changed; unemployed; not done/missing |
| WPAQ change in level of responsibility at work | Employed, unchanged; employed, changed; unemployed; not done/missing |
| CTSQ expectations of therapy | Below median score; median score or above; not done/missing |
| CTSQ feelings about side effects | Below median score; median score or above; not done/missing |
| CTSQ satisfaction with therapy | Below median score; median score or above; not done/missing |
| EQ-5D UK index score | Below median score; median score or above; not done/missing |
| EQ-5D VAS score | Below median score; median score or above; not done/missing |
| ECOG PS at study entry | Asymptomatic; symptomatic, ambulatory; symptomatic, less than 50% in bed or chair; symptomatic, 50–100% in bed or chair; not done/missing |

^a^Apart from sex, the referent was selected as the category expected to have the highest mortality rate. All covariates were modelled as categorical variables. There was little variation in race; therefore, this variable was not evaluated as a covariate. Covariates were identified as predictors of mortality at a significance level of 0.10 with ipilimumab therapy (3 indicator variables) forced in the model. Among the 25 covariates evaluated in the models, 6 were selected by the analysis: ECOG performance status at study entry, LDH status, EORTC QLQ-30 quality of life, prior therapy, sex, and WPAQ change in level of responsibility at work.

CTSQ, Cancer Therapy Satisfaction Questionnaire; ECOG PS, Eastern Cooperative Oncology Group performance status; EORTC, European Organisation for Research and Treatment of Cancer; EQ-5D, EuroQol-5D; LDH, lactate dehydrogenase; OS, overall survival; VAS, visual analog scale; WPAI-GH, Work Productivity and Activity Impairment-General Health; WPAQ, Work-related Physical Activity Questionnaire.

**Table A.2** Ipilimumab dosing

|  | **All IPI-treated patients^a^**  **(n=1151)** | **Patients who completed IPI therapy^b^**  **(n=1137)** |
| --- | --- | --- |
| IPI infusions received | | |
| 1 | 105 (9) | 97 (9) |
| 2 | 154 (13) | 152 (13) |
| 3 | 170 (15) | 166 (15) |
| 4 | 719 (62) | 719 (63) |
| 5 | 3 (˂ 1) | 3 (˂1) |
| Number of infusions per patient | | |
| Mean (SD) | 3.3 (1.0) | 3.3 (1.0) |
| Median (range) | 4 (1–5) | 4 (1–5) |

^a^Includes patients who received ipilimumab therapy (completed or ongoing) at any time during the study period (IPI-noOther, IPI-Other, and Other-IPI).

^b^Includes patients who received all 4 recommended infusions of ipilimumab or those who permanently discontinued ipilimumab before receiving all 4 infusions.

IPI, ipilimumab; SD, standard deviation.

**Table A.3** Summary of treatment-related adverse events in patients who received ipilimumab therapy at any time during the study period (includes groups 1, 2, and 4)

|  | **All IPI-treated patients (n=1151)^a^, on-study^b^** | |
| --- | --- | --- |
|  | **Any grade, n (%)^c^** | **Grade ≥3, n (%)** |
| Any TRAE | 756 (66) | 294 (26) |
| Diarrhoea | 242 (21) | 67 (6) |
| Fatigue | 179 (16) | 11 (1) |
| Rash | 136 (12) | 14 (1) |
| Nausea | 120 (10) | 7 (1) |
| Pruritus | 104 (9) | 6 (1) |
| Colitis | 77 (7) | 54 (5) |
| Vomiting | 63 (5) | 6 (1) |
| Decreased appetite | 60 (5) | 2 (<1) |
| Headache | 60 (5) | 7 (1) |

^a^Includes patients who received ipilimumab therapy at any time during the study period (IPI-noOther, IPI-Other, and Other-IPI).

^b^From first dose of ipilimumab until discontinuation from study or end of study, whichever occurred first.

^c^Occurring in ≥5% of patients.

IPI, ipilimumab; TRAE, treatment-related adverse event.

**Table A.4** Summary of immune-related adverse events in patients who received ipilimumab therapy at any time during the study period (includes groups 1, 2, and 4)

|  | **All IPI-treated patients^a^** | | |
| --- | --- | --- | --- |
|  | **On study^b^  (n=1151)** | **Early onset^c^  (n=1151)** | **Late onset^d^  (n=653)** |
| Any immune-related adverse event | | | |
| Any grade | 569 (49) | 550 (48) | 65 (10) |
| Grade ≥3 | 211 (19) | 211 (18) | 20 (3) |
| Gastrointestinal | | | |
| Any grade | 312 (27) | 295 (26) | 27 (4) |
| Grade ≥3 | 127 (11) | 122 (11) | 7 (1) |
| Skin | | | |
| Any grade | 270 (23) | 251 (22) | 36 (6) |
| Grade ≥3 | 24 (2) | 21 (2) | 4 (1) |
| Endocrine | | | |
| Any grade | 85 (7) | 72 (6) | 14 (2) |
| Grade ≥3 | 33 (3) | 30 (3) | 4 (1) |
| Hepatic | | | |
| Any grade | 65 (6) | 61 (5) | 5 (1) |
| Grade ≥3 | 31 (3) | 29 (3) | 2 (˂ 1) |
| Neurological | | | |
| Any grade | 27 (2) | 22 (2) | 6 (1) |
| Grade ≥3 | 4 (˂1) | 4 (˂1) | 0 |
| Other | | | |
| Any grade | 22 (2) | 14 (1) | 8 (1) |
| Grade ≥3 | 10 (1) | 8 (1) | 2 (˂1) |

^a^Includes patients who received ipilimumab therapy at any time during the study period (IPI-noOther, IPI-Other, and Other-IPI).

^b^From first dose of ipilimumab until discontinuation from study or end of study, whichever came first.
^c^Occurring between the first dose and 90 days after the last dose of ipilimumab therapy.
^d^Occurring 90 days after the last dose of ipilimumab; the denominator is the number of ipilimumab-treated patients who were in the post-treatment phase of follow-up.

IPI, ipilimumab.

**Table A.5** Incidence rate of treatment-related adverse events of grade ≥3 in patients who received ipilimumab therapy at any time during the study period^a^ (includes groups 1, 2, and 4)

|  | **Incidence rate per 100 person-years^b^ (95% CI)** | | |
| --- | --- | --- | --- |
|  | **Year 1  (n=1151)** | **Year 2  (n=271)** | **Cumulative  (n=1151)** |
| **Gastrointestinal**  Grade 3/4  Grade 5  Grade unknown  Serious | **10.9 (9.4–12.5)**  31.9 (27.5–36.8)  0.5 (0.1–1.4)  1.8 (0.9–3.1)  12.4 (10.5–14.5) | **3.0 (1.1–6.6)**  3.0 (1.1–6.6)  0 (NA–1.9)  0 (NA–1.9)  1.5 (0.3–4.4) | **7.5 (6.5–8.5)**  22.1 (19.1–25.4)  0.3 (0.1–0.9)  1.1 (0.6–2.1)  8.3 (7.1–9.7) |
| **Skin**  Grade 3/4  Grade 5  Grade unknown  Serious | **3.0 (2.1–4.1)**  4.2 (2.8–6.2)  0 (NA–0.6)  1.8 (0.9–3.1)  0.8 (0.3–1.9) | **0 (NA–1.9)**  0 (NA–1.9)  0 (NA–1.9)  0 (NA–1.9)  0 (NA–1.9) | **2.0 (1.4–2.7)**  2.8 (1.8–4.1)  0 (NA–0.4)  1.2 (0.6–2.1)  0.5 (0.2–1.2) |
| **Hepatobiliary**  Grade 3/4  Grade 5  Grade unknown  Serious | **3.1 (1.8–4.8)**  3.1 (1.8–4.8)  0 (NA–0.6)  0 (NA–0.6)  1.8 (0.9–3.2) | **0 (NA–1.9)**  0 (NA–1.9)  0 (NA–1.9)  0 (NA–1.9)  0 (NA–1.9) | **2.0 (1.2–3.1)**  2.0 (1.2–3.1)  0 (NA–0.4)  0 (NA–0.4)  1.2 (0.6–2.1) |
| **Endocrine**  Grade 3/4  Grade 5  Grade unknown  Serious | **3.0 (2.1–4.1)**  5.7 (4.0–8.0)  0 (NA–0.6)  0.3 (0–1.2)  5.2 (3.6–7.4) | **0 (NA–1.9)**  0 (NA–1.9)  0 (NA–1.9)  0 (NA–1.9)  0 (NA–1.9) | **2.0 (1.4–2.7)**  3.8 (2.6–5.3)  0 (NA–0.4)  0.2 (0–0.8)  3.5 (2.4–4.9) |
| **Nervous system**  Grade 3/4  Grade 5  Grade unknown  Serious | **1.6 (1.1–2.3)**  3.9 (2.5–5.8)  0.2 (0–0.9)  0.8 (0.3–1.9)  1.4 (0.8–2.2) | **0.5 (0–2.8)**  0.5 (0–2.8)  0.5 (0–2.8)  0 (NA–1.9)  0.5 (0–2.8) | **1.1 (0.7–1.5)**  2.6 (1.7–3.9)  0.1 (0–0.6)  0.5 (0.2–1.2)  0.9 (0.6–1.5) |

^a^From first dose of ipilimumab until discontinuation from study or end of study, whichever came first.
^b^Calculated by dividing the number of events by overall total exposure (the sum of the exposure time for all ipilimumab-treated patients) during the specified time at risk. The resulting incidence rate was multiplied by 100 to express the rate per 100 person-years. Poisson rate CIs were calculated for the incidence rates.

NA, not available.

**Table A.6** Tumor response

| **Tumor response^a^** | **IPI-treated cohort** | | **Non-IPI–treated cohort** | |
| --- | --- | --- | --- | --- |
|  | **IPI-noOther (n=780), n (%)** | **IPI-Other (n=314), n (%)** | **Other-Other (n=205), n (%)** | **Other-IPI (n=57), n (%)** |
| Disease control rate^b^ | 124 (16) | 62 (20) | 32 (16) | 4 (7) |
| Complete response | 17 (2) | 9 (3) | 6 (3) | 1 (2) |
| Partial response | 13 (2) | 16 (5) | 11 (5) | 0 |
| Stable disease | 94 (12) | 37 (12) | 15 (7) | 3 (5) |
| Progressive disease | 503 (64) | 237 (75) | 119 (58) | 50 (88) |
| Indeterminate | 14 (2) | 6 (2) | 5 (2) | 1 (2) |
| Missing | 2 (<1) | 0 | 0 | 0 |
| Not applicable^c^ | 137 (18) | 9 (3) | 49 (24) | 2 (4) |

^a^Tumor response was based on the last tumor assessment record with a non-missing assessment date during the on-study period. Tumor response was determined by investigators using various response criteria (i.e., the WHO criteria, RECIST, or other response criteria).

^b^The sum of complete response, partial response, and stable disease rates.

^c^There was no tumor response data during the study period.

RECIST, Response Evaluation Criteria in Solid Tumors; WHO, World Health Organization.

**Table A.7** Patient-reported outcome completion rates^a^

|  | **All prospective patients**  **(N=1356)** | **IPI-treated cohort** | | **Non-IPI–treated cohort** | |
| --- | --- | --- | --- | --- | --- |
|  |  | **IPI-noOther  (n=780)** | **IPI-Other  (n=314)** | **Other-Other (n=205)** | **Other-IPI  (n=780)** |
| Baseline^b^ | 1049/1356 (77) | 612/780 (78) | 245/314 (78) | 153/205 (75) | 39/57 (68) |
| Month 3 | 729/944 (77) | 355/477 (74) | 229/286 (80) | 100/127 (79) | 45/54 (83) |
| Month 6 | 407/593 (69) | 169/246 (69) | 153/222 (69) | 54/81 (67) | 31/44 (70) |
| Month 9 | 289/417 (69) | 114/167 (68) | 108/160 (68) | 44/62 (71) | 23/28 (82) |
| Month 12 | 188/304 (62) | 71/118 (60) | 78/125 (62) | 28/43 (65) | 11/18 (61) |
| Month 15 | 146/247 (59) | 59/103 (57) | 63/103 (61) | 16/29 (55) | 8/12 (67) |
| Month 18 | 135/221 (61) | 54/94 (57) | 56/90 (62) | 16/26 (62) | 9/11 (82) |
| Month 21 | 128/194 (66) | 59/88 (67) | 49/77 (64) | 13/21 (62) | 7/8 (88) |
| Month 24 | 109/174 (63) | 58/83 (70) | 40/66 (61) | 9/19 (47) | 2/6 (33) |
| Month 27 | 95/155 (61) | 47/79 (59) | 33/54 (61) | 10/17 (59) | 5/5 (100) |
| Month 30 | 85/146 (58) | 42/78 (54) | 29/46 (63) | 10/17 (59) | 4/5 (80) |
| Month 33 | 85/140 (61) | 46/76 (61) | 26/44 (59) | 11/16 (69) | 2/4 (50) |
| Month 36 | 70/87 (80) | 39/52 (75) | 21/23 (91) | 8/9 (89) | 2/3 (67) |

^a^Calculated using the number of patients with non-missing patient-reported outcome data at baseline and data from post-baseline visit, divided by the number of patients in the study at each respective time point.

^b^Completion rate based on patients having any baseline data with no post-baseline data requirement.

**Figure A.1** Study design.


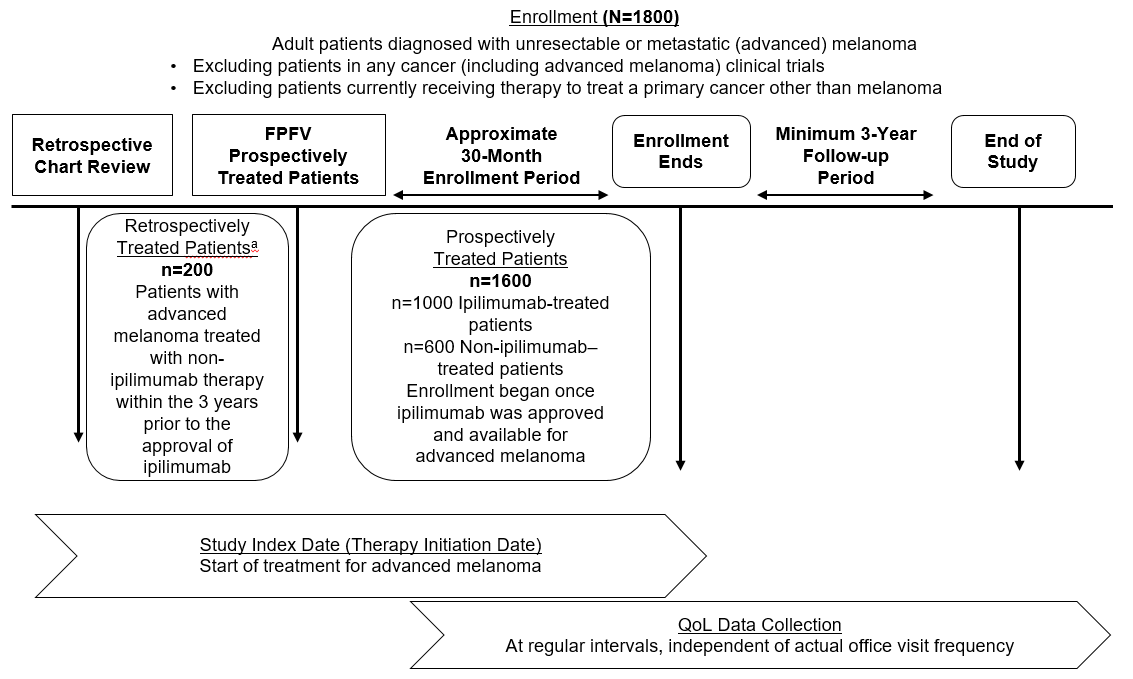


^a^Received at least one prior therapy for unresectable or metastatic melanoma. FPFV, first patient first visit.

**Figure A.2** OS in the ipilimumab-treated and non–ipilimumab-treated cohorts. *P* value was estimated from type 3 Wald test from the Cox model. OS, overall survival.


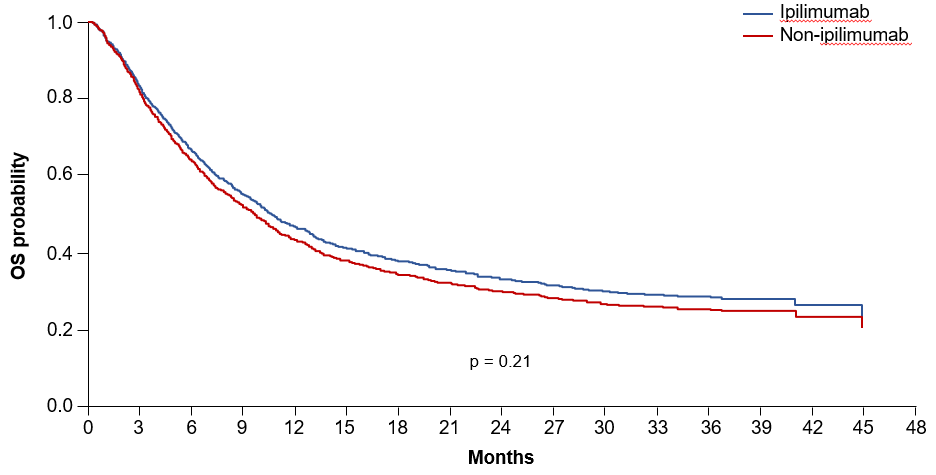


**Figure A.3** OS in (A) previously treated (*n* = 1064) and (B) treatment-naive (*n* = 292) patients. *P* values were estimated from type 3 Wald test from the Cox model. OS, overall survival.


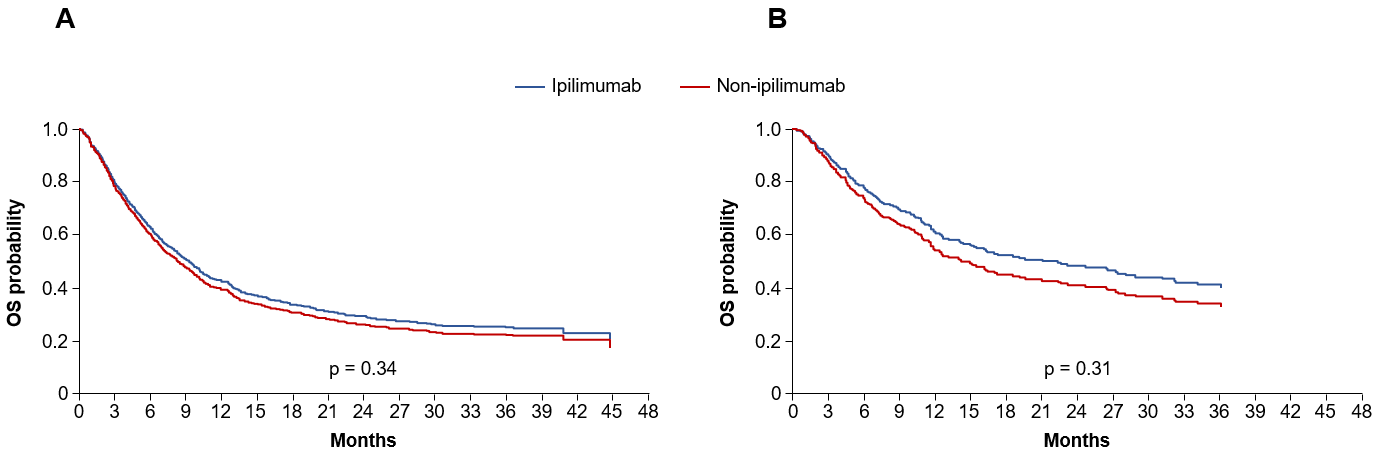


**Figure A.4** Patient-reported outcomes in three symptom scales (fatigue, nausea and vomiting, and pain), six single-item symptom scores (dyspnea, insomnia, appetite loss, constipation, diarrhea, and financial difficulties), and five functional scales (physical, role, cognitive, social, and emotional functioning).

**A**


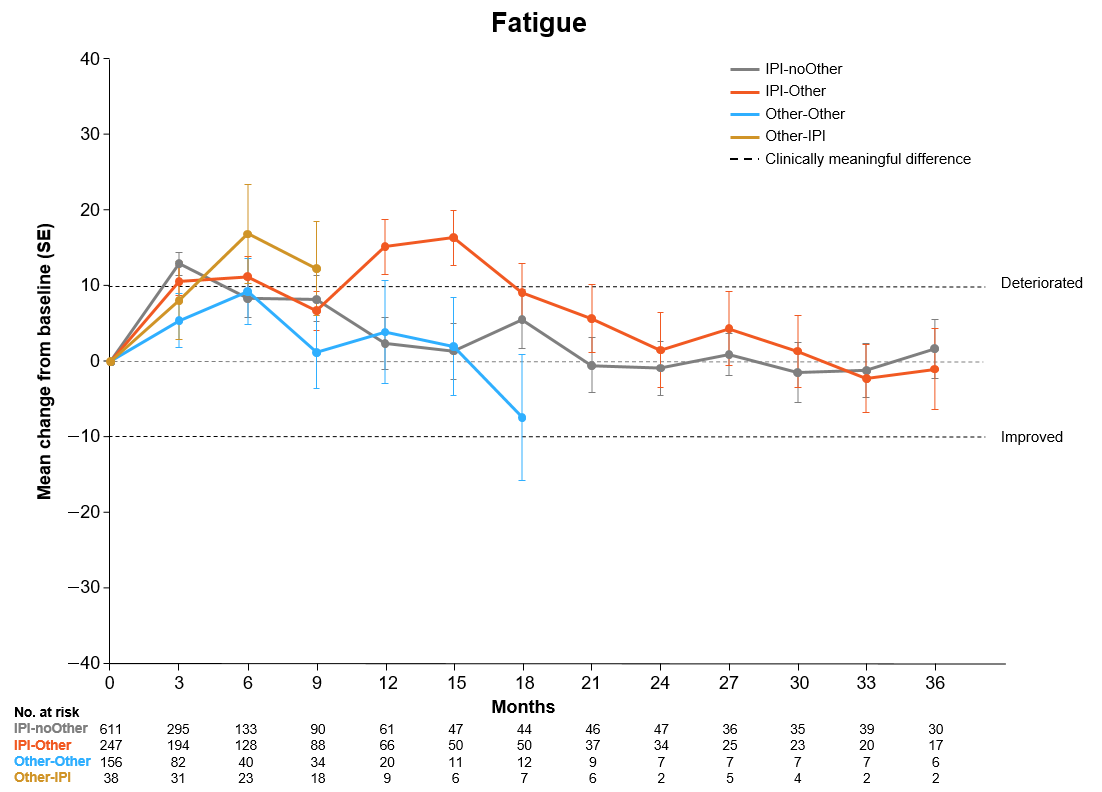


**B**


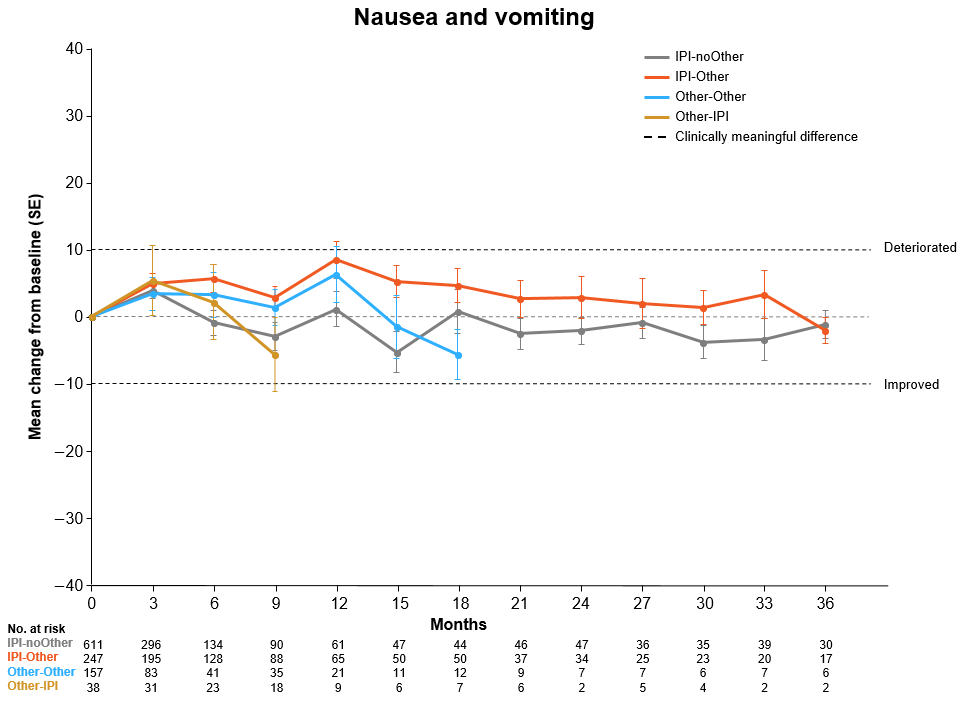


**C**


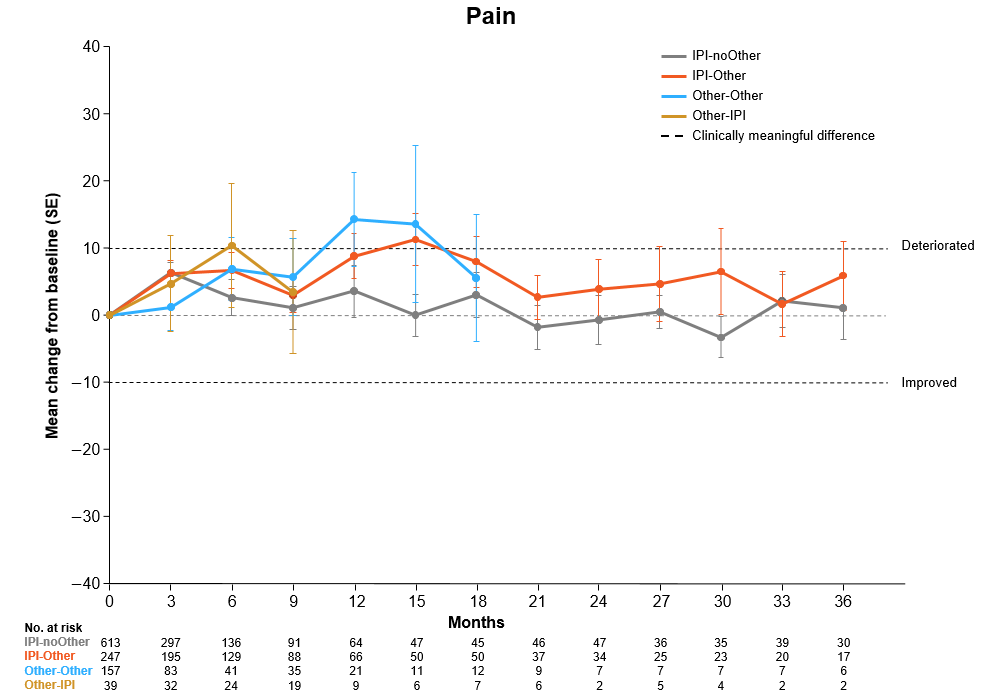


**D**


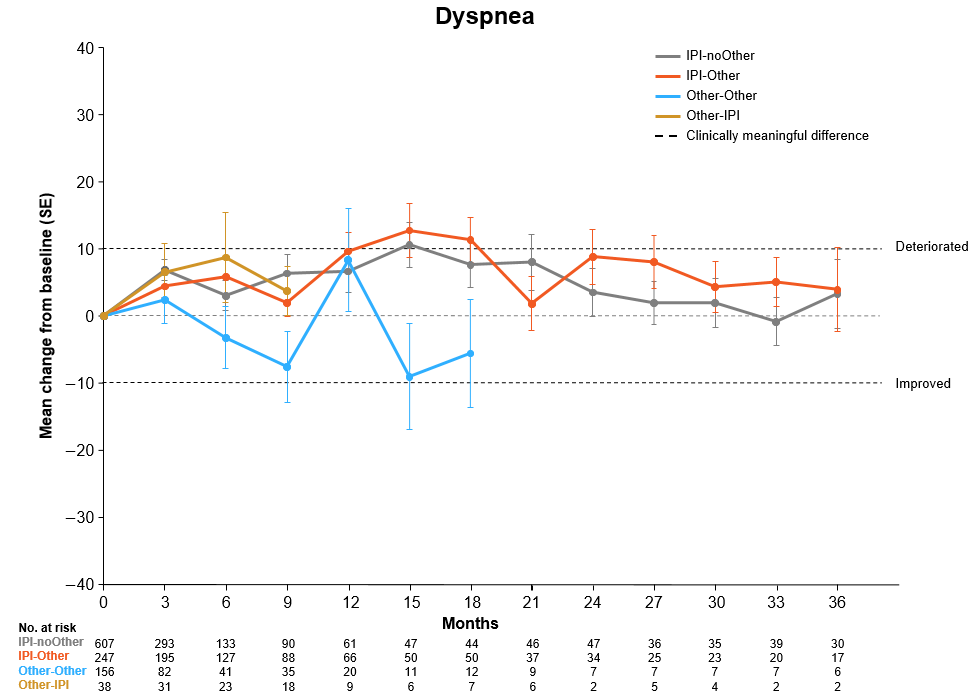


**E**


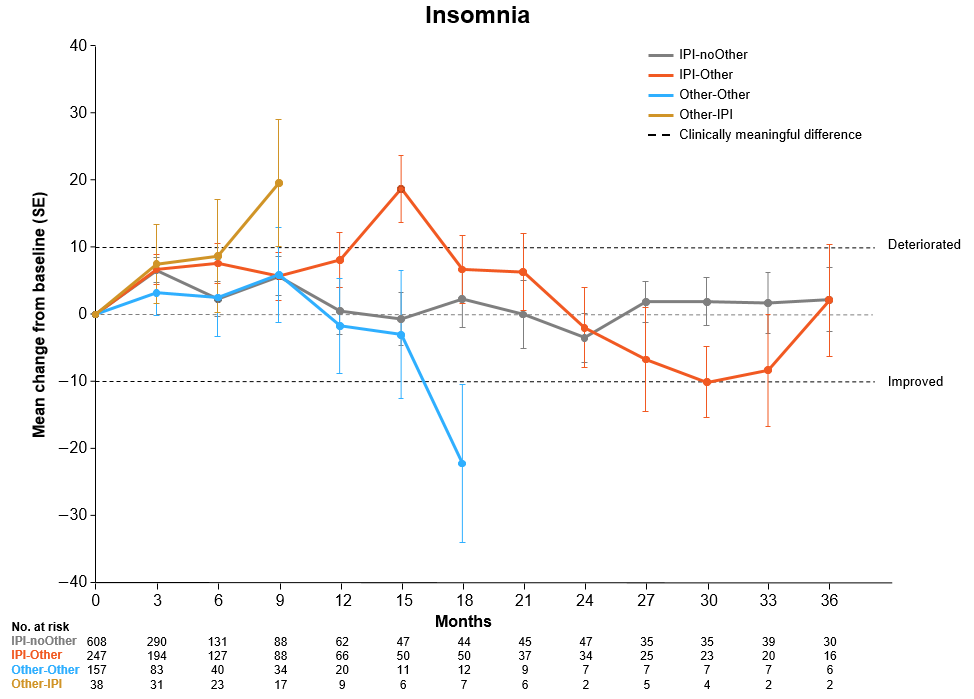


**F**


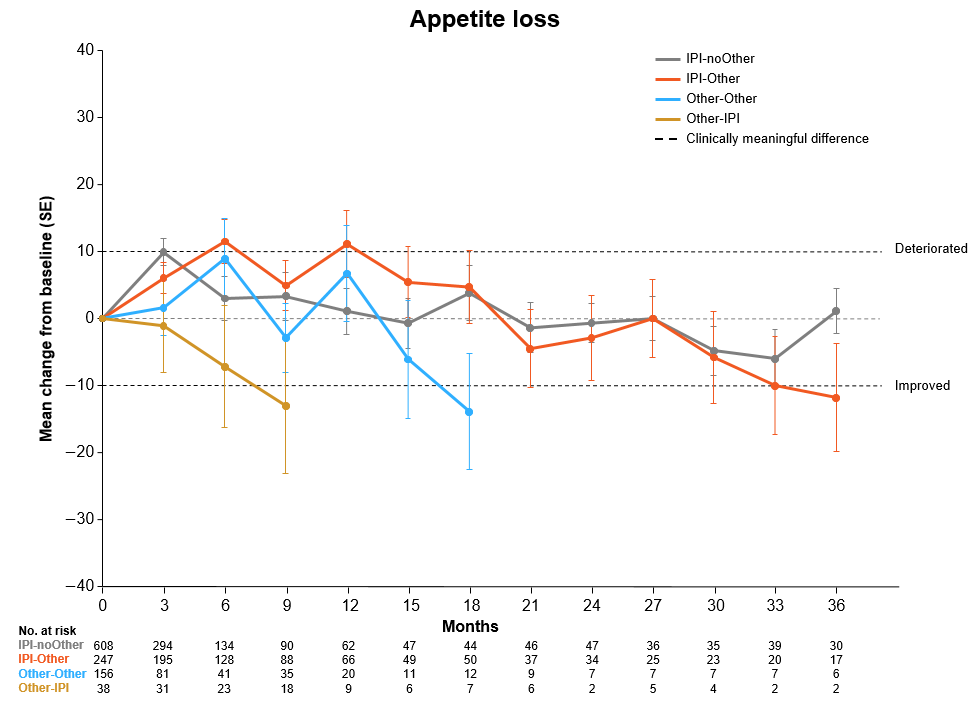


**G**


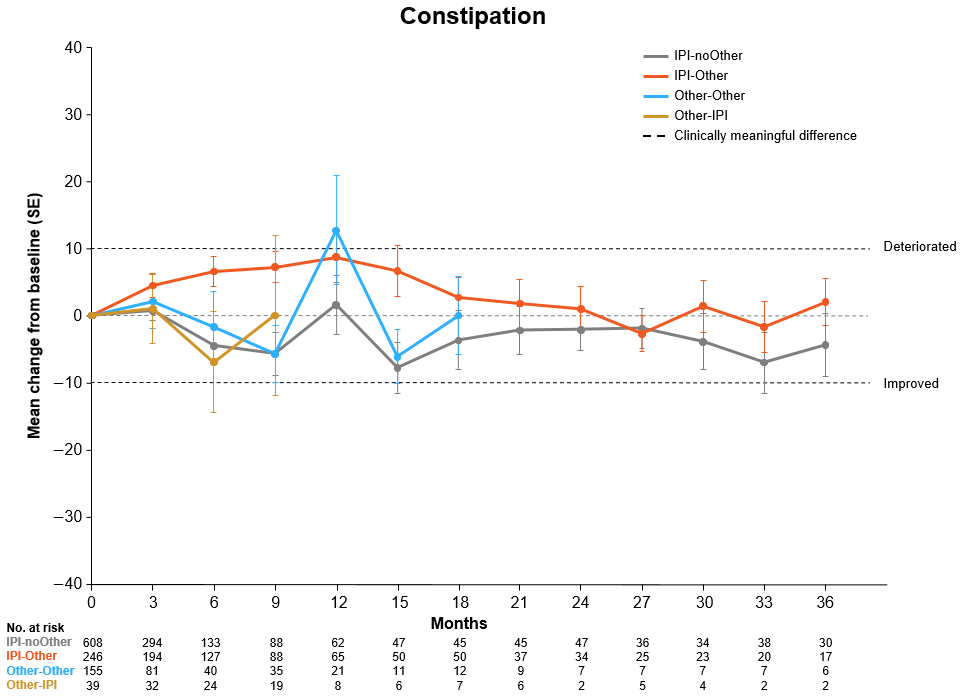


**H**


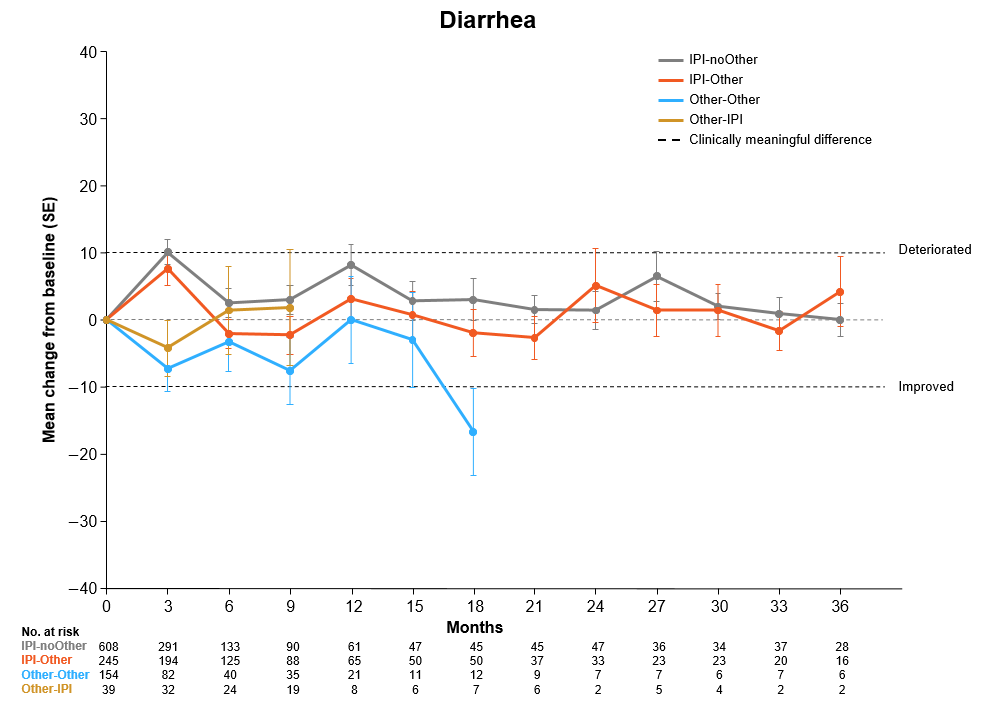


**I**


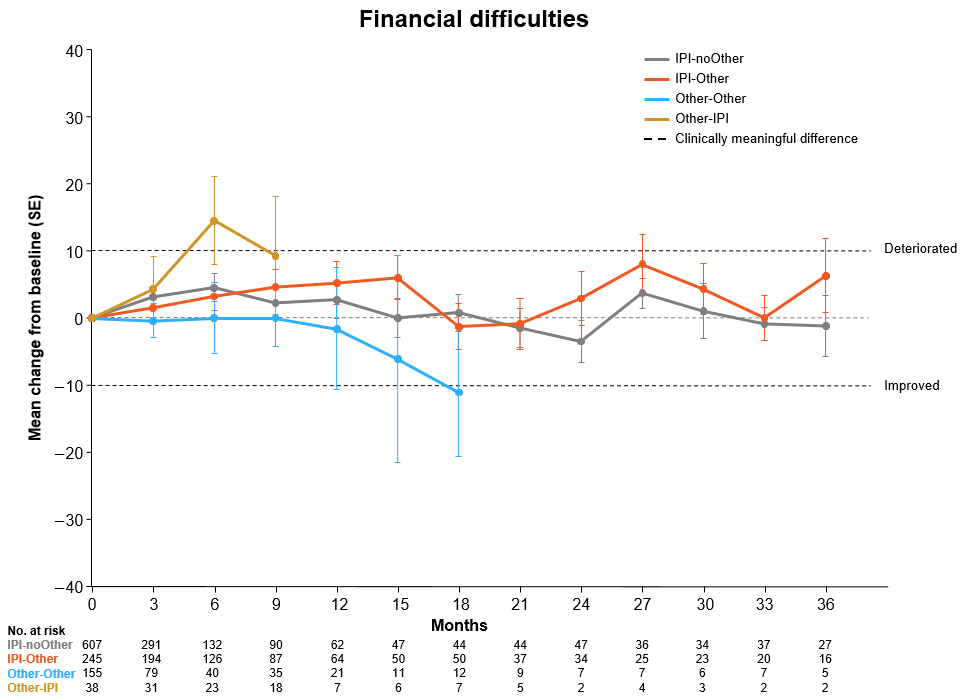


**J**


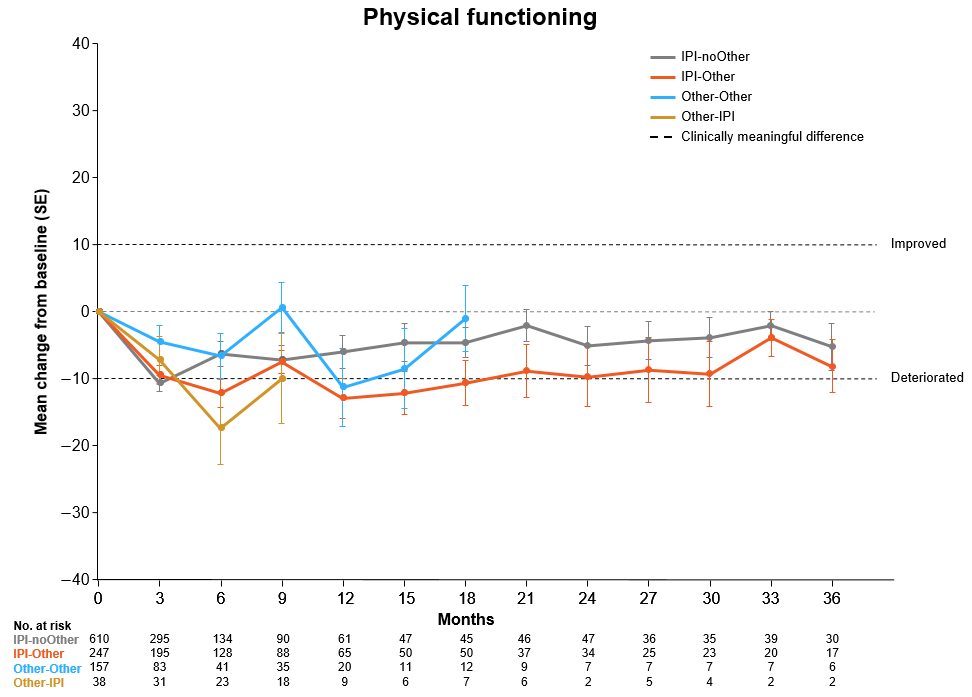


**K**


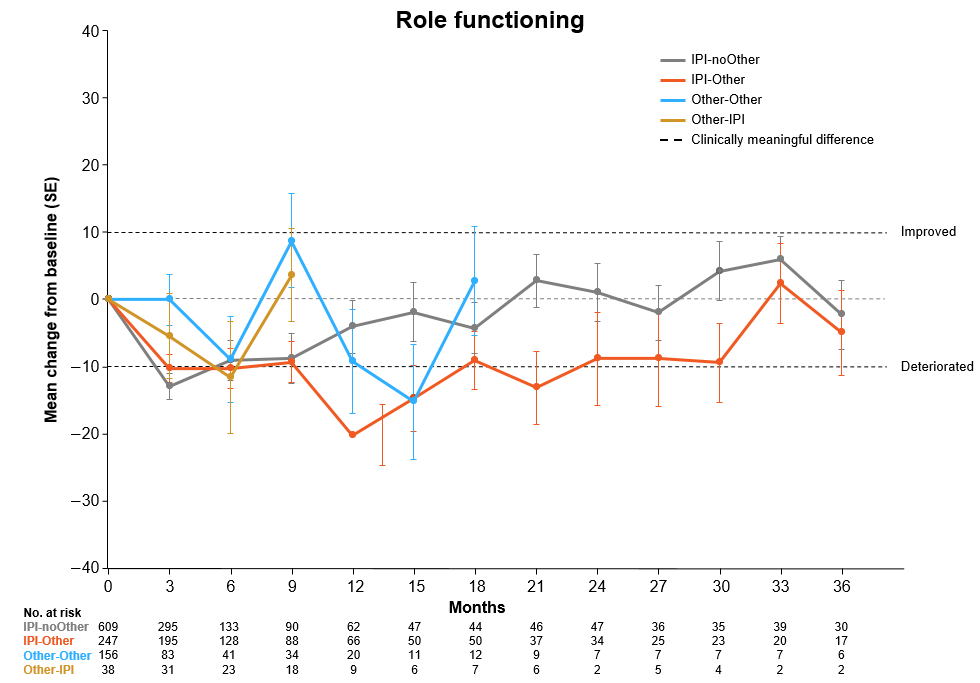


**L**


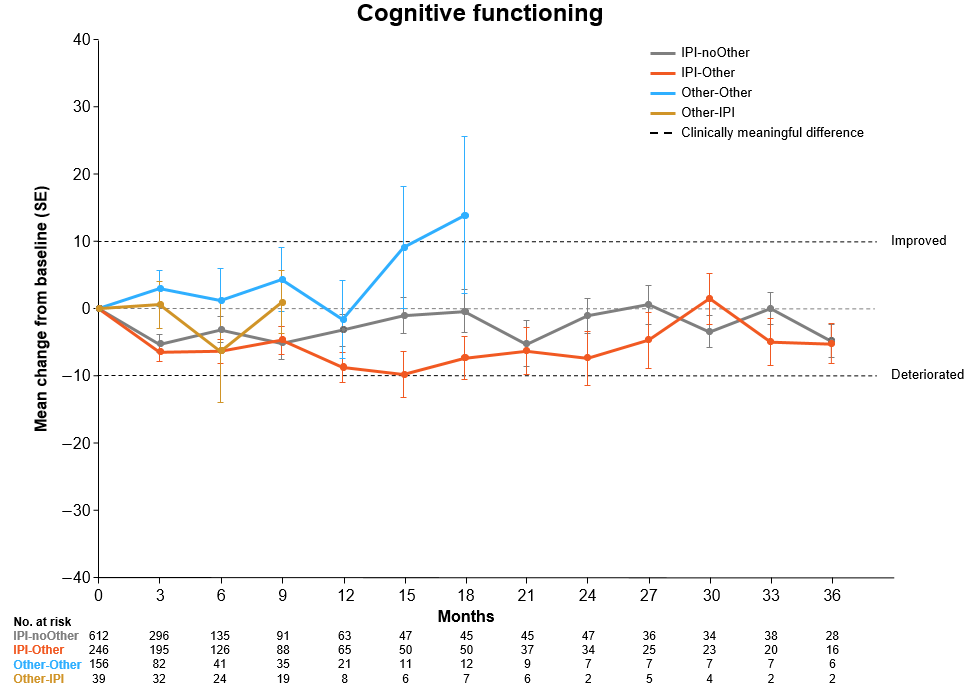


**M**


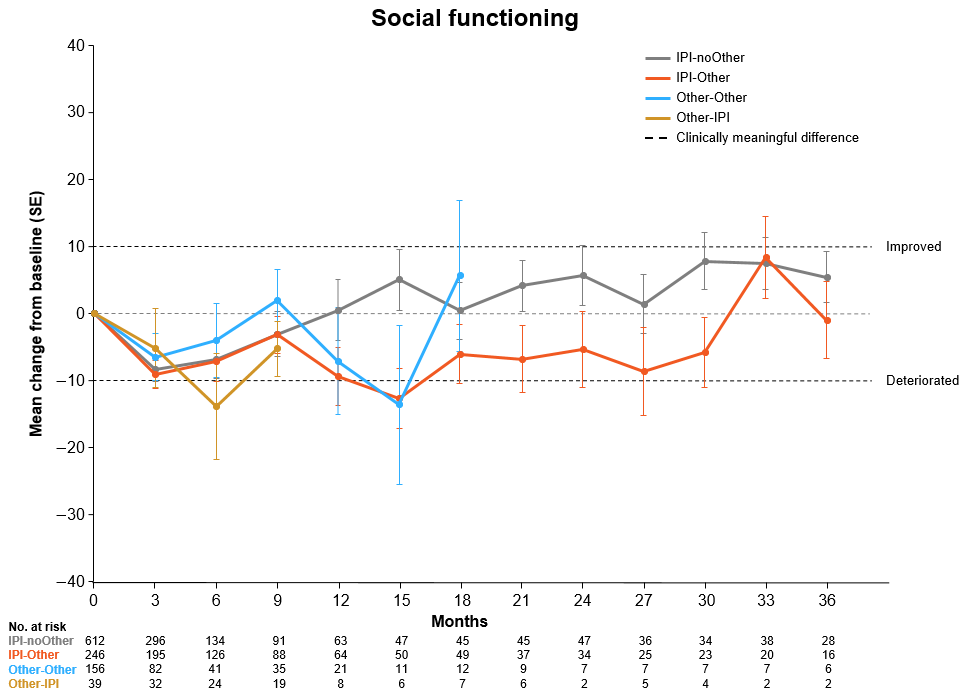


**N**


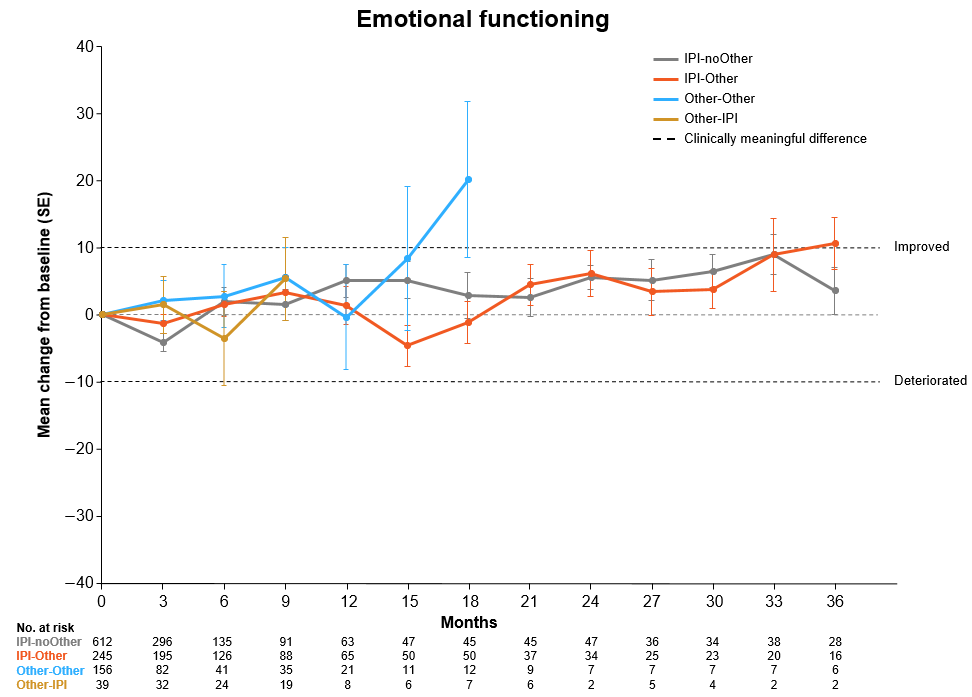

Supplement: Supplementary file 1 — Additional file 1: Supplement. Table A.1. Covariates evaluated in the multivariate Cox regression models for OS probabilities. Table A.2. Ipilimumab dosing. Table A.3. Summary of treatment-related adverse events in patients who received ipilimumab therapy at any time during the study period (includes groups 1, 2, and 4). Table A.4. Summary of immune-related adverse events in patients who received ipilimumab therapy at any time during the study period (includes groups 1, 2, and 4). Table A.5. Incidence rate of treatment-related adverse events of grade ≥ 3 in patients who received ipilimumab therapy at any time during the study period (includes groups 1, 2, and 4). Table A.6. Tumor response. Table A.7. Patient-reported outcome completion rates. Fig. A.1. Study design. Fig. A.2. OS in the ipilimumab-treated and non-ipilimumab-treated cohorts. P value was estimated from type 3 Wald test from the Cox model. OS Overall survival. Fig. A.3. OS in (A) previously treated (n = 1064) and (B) treatment-naive (n = 292) patients. P values were estimated from type 3 Wald test from the Cox model. OS overall survival. Fig. A.4. Patient-reported outcomes in three symptom scales (fatigue, nausea and vomiting, and pain), six single-item symptom scores (dyspnea, insomnia, appetite loss, constipation, diarrhea, and financial difficulties), and five functional scales (physical, role, cognitive, social, and emotional functioning) [file 12885_2021_8032_MOESM1_ESM.docx]
